# Accelerated immunosenescence in SLE: current evidence and clinical translation

**DOI:** 10.3389/fimmu.2026.1830804

**Published:** 2026-06-17

**Authors:** Qingshuang Li, Wei Zhang, Yulan Chen, Jing Du

**Affiliations:** 1 Department of Clinical Laboratory, Peking University Shenzhen Hospital, Shenzhen, Guangdong, China; 2 Department of Experimental Research, South China Hospital, Medical School, Shenzhen University, Shenzhen, China; 3 Department of Rheumatology and Immunology, Shenzhen People’s Hospital (The Second Clinical Medical College, Jinan University; The First Affiliated Hospital, Southern University of Science and Technology), Shenzhen, Guangdong, China

**Keywords:** systemic lupus erythematosus (SLE), immunosenescence, inflammaging, senescence-associated secretory phenotype (SASP), immune clock, epigenetic aging, clinical translation, immune juvenation

## Abstract

**Background:**

Patients with systemic lupus erythematosus (SLE) often develop age-related adverse outcomes at a young age, raising the possibility that immune-aging processes may interact with chronic immune activation, treatment exposure, and accumulated damage in shaping long-term disease burden. Although immune-age acceleration has also been reported in rheumatoid arthritis and chronic viral infections (e.g., HIV/CMV), SLE is particularly well suited as a human model of inflammation-driven immune aging because of its typical onset in young women and the convergence of aging signatures across clinical, cellular, and molecular levels.

**Main body:**

This review synthesizes evidence for accelerated immunosenescence in SLE across systemic manifestations, immune cell remodeling, and molecular senescence markers. Clinically, SLE is linked to premature cardiovascular disease, frailty, cognitive impairment, severe infections, and reduced vaccine responsiveness. Immunologically, senescent-like immune subsets (including terminally differentiated T cells, age-associated/double-negative B cells, dysfunctional NK cells, and senescence-like myeloid cells) expand prematurely. Molecular features include inflammaging-like cytokine patterns, telomere attrition, and epigenetic age acceleration. We also discuss a translational framework for quantifying immunosenescence using immunophenotyping, telomere/epigenetic clocks, and composite immune clocks, with potential applications in risk stratification, treatment decisions, and monitoring immune-reprogramming strategies.

**Conclusions:**

Accelerated immunosenescence provides a biologically plausible and clinically relevant framework for understanding SLE heterogeneity across systemic, cellular, and molecular levels. However, immune-aging measures should currently be regarded as candidate research biomarkers rather than validated clinical decision tools. Prospective SLE-specific studies are needed to establish their predictive value, disease specificity, and clinical utility.

## 1 Introduction

Systemic lupus erythematosus (SLE) is a highly heterogeneous systemic autoimmune disease with complex etiology that can affect multiple organ systems, characterized by chronic progression and recurrent episodes (1, 2). It represents a major cause of long-term morbidity among women of childbearing age worldwide (3). While predominantly affecting young women, clinical observations consistently indicate that SLE patients exhibit marked immune dysfunction beyond their physiological age, including increased susceptibility to infections (4, 5), suboptimal vaccine responses (6, 7), premature atherosclerosis (8), and cumulative multisystem damage (3). These phenomena, which mirror age-related immune decline, consistent with premature aging, cannot be fully explained by the traditional immune hyperactivation mode (9). This has led researchers to recognize that the immune system in SLE may exist in an abnormal state characterized by both persistent activation and accelerated immune aging (immunosenescence) (10).

Immunosenescence, a core concept in immunology, was initially proposed to describe age-related immune decline (11, 12). Its theoretical origin can be traced to the “immunologic theory of aging” proposed by Roy Walford in the late 1960s, which linked increased susceptibility to infections and cancer in older adults to progressive immune dysfunction (13). It has since expanded to encompass non-age-dependent immune aging driven by inflammation, metabolic stress, and chronic immune stimulation. In the 1990s and early 2000s, Franceschi and colleagues further advanced this concept through the framework of inflammaging, emphasizing chronic low-grade inflammation as a central mechanism underlying immune remodeling and biological aging (14). From this perspective, immunosenescence is not confined to the elderly but may represent a key pathogenic substrate for many chronic immune-mediated diseases. Against this backdrop, the intersection between SLE and immunosenescence has become a major focus of research over the past two decades (15, 16).

In recent years, immunosenescence has evolved from an abstract concept to a quantifiable entity in the form of immune age and immune clocks based on immunophenotyping, epigenetic markers, and inflammatory profiles (14, 17, 18). While multiple studies have reported evidence linking SLE to immunosenescence at various levels, most investigations remain fragmented across systemic, cellular, and molecular domains, with substantial methodological heterogeneity (9, 19–21). As a result, a unified conceptual and analytical framework for interpreting accelerated immunosenescence in SLE pathogenesis and disease progression is still lacking (22).

In this review, immunosenescence refers to age-associated remodeling of the immune system, including reduced immune renewal capacity, contraction of naïve lymphocyte pools, restriction of antigen receptor diversity, accumulation of terminally differentiated or senescent immune cells, and impaired immune surveillance. This concept should be distinguished from inflammaging, treatment-related toxicity, and accumulated damage. Inflammaging describes chronic, low-grade systemic inflammation associated with aging and may amplify immune dysfunction, but it does not fully capture the cellular and repertoire-level remodeling implied by immunosenescence. Treatment-related toxicity refers to immune or organ consequences attributable to glucocorticoids, immunosuppressants, biologics, or cellular therapies, whereas accumulated damage reflects irreversible organ injury resulting from disease activity, comorbidities, and treatment exposure. These processes may interact in SLE, but they are conceptually distinct and should not be treated as interchangeable (23).

**Figure 1:**
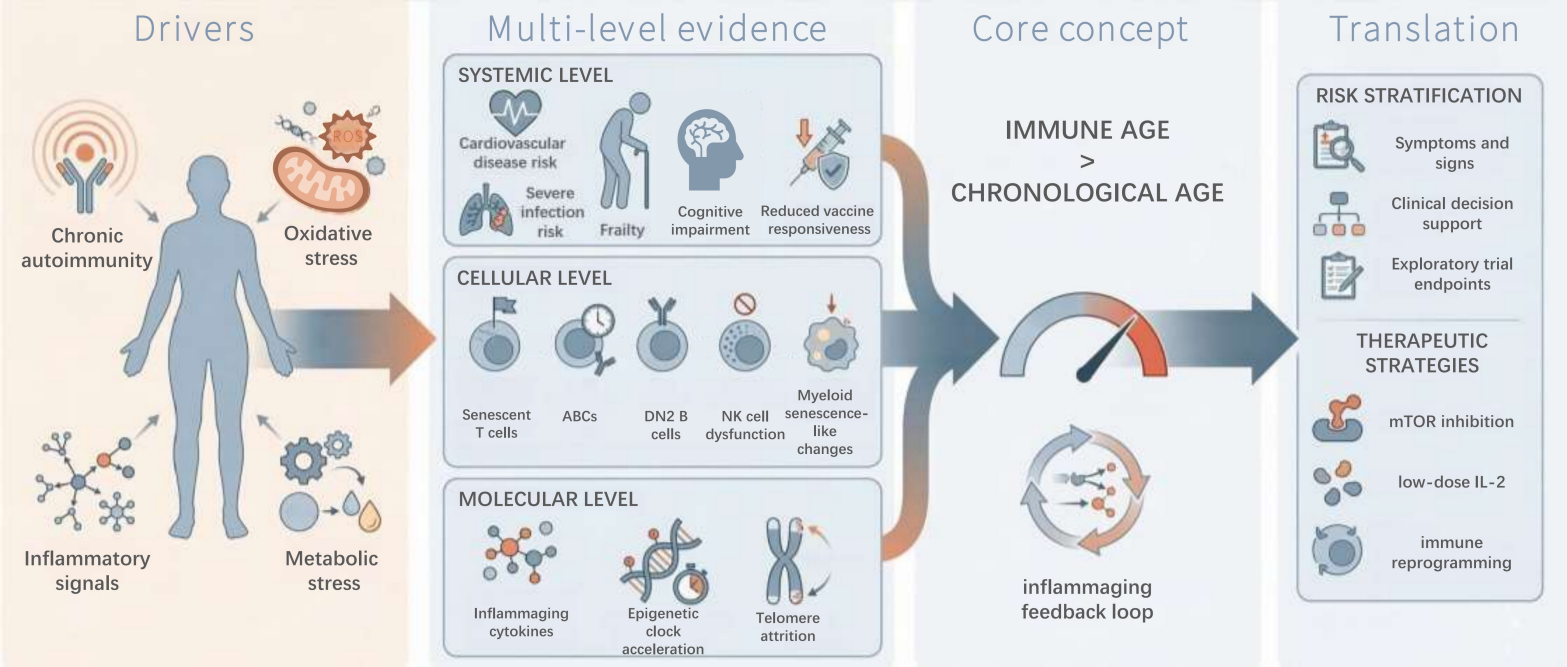
Schematic representation of accelerated immunosenescence in SLE. Accelerated immunosenescence is presented here as a unifying framework for understanding SLE across systemic, cellular and molecular scales. Rather than a single linear pathway, the model highlights convergence of chronic autoimmune and inflammatory stress into coexisting age-associated immune alterations, with potential consequences for clinical vulnerability and therapeutic stratification. The figure is intended to frame immune age as a biologically meaningful dimension of SLE, distinct from chronological ageing and relevant to translational decision-making. Arrows denote conceptual convergence and proposed relationships rather than established causal sequence.

On this basis, this review proposes an immunosenescence-centered framework integrating evidence of accelerated immune aging in SLE across systemic, cellular, and molecular dimensions (**Figure 1**).

## 2 Multi-level evidence for accelerated immunosenescence

### 2.1 Systemic level

In this review, systemic manifestations refer to clinically observable phenotypes that may reflect immune-system aging at the organismal level, whereas age-related comorbidities refer to diseases that increase with aging and may be promoted by SLE-related inflammation, treatment exposure, and accumulated damage. Therefore, cardiovascular disease, frailty, severe infection, impaired vaccine responsiveness, and cognitive impairment are discussed here as clinical phenotypes consistent with accelerated immunosenescence, rather than as disease-specific consequences of immune aging.

Although SLE predominantly affects young and middle-aged adults (1, 2), its long-term clinical trajectory is marked by several clinical phenotypes and comorbidities that are typically associated with advanced age, including cardiovascular disease (24), osteoporosis (25), frailty (26), malignancy (27), and recurrent severe infections (4, 28). These systemic manifestations are not merely comorbidities but represent clinical correlates of accelerated immunosenescence.

From an immunological perspective, many of these conditions arise as consequences of chronic immune activation, impaired immune surveillance, and sustained inflammatory burden (“inflammaging”), which together mirror the immune profile observed in elderly individuals. Thus, SLE provides a unique human model of premature immune aging, in which aging-related outcomes emerge decades earlier than expected.

#### 2.1.1 Premature atherosclerosis and cardiovascular disease

Multiple cohort and imaging studies have demonstrated that SLE patients develop significant coronary atherosclerotic plaques and vascular calcification at much younger ages than age-matched healthy controls, even after adjustment for traditional cardiovascular risk factors (29, 30).

Importantly, atherosclerosis is increasingly recognized as an immune-mediated disorder driven by chronic inflammation, dysregulated adaptive immunity, and impaired immune surveillance - hallmark features of immunosenescence (31, 32). In the general population, such immune-driven vascular pathology typically manifests after the sixth decade of life (33). In contrast, SLE patients accumulate a comparable atherosclerotic burden within 10–20 years of disease onset, often during reproductive age (30, 34).

The so-called second-hit hypothesis further supports this concept, proposing that chronic autoimmune activation in SLE constitutes the first hit, whereas age-associated comorbidities such as cardiovascular disease, osteoporosis, and malignancy represent downstream consequences of accelerated immune aging (35). As a result, patients in their 30s-40s exhibit a multimorbidity profile that resembles that of elderly individuals. From an immunosenescence perspective, this striking temporal shift represents a prototypical example of “clinical age mismatch,” in which immune system aging precedes chronological aging and translates into premature cardiovascular disease.

#### 2.1.2 Frailty, cognitive impairment, and aging-related phenotypes

Frailty Index (FI) constructed using deficit accumulation models has shown that a substantial proportion of adult SLE patients-including non-elderly women-already meet criteria for frailty or prefrailty (36–38). The FI scores are strongly associated with mortality, hospitalization risk, and self-reported poor health status. Frailty is increasingly recognized as a clinical surrogate of biological aging and immune decline, closely linked to chronic inflammation and immunosenescence (39).

Longitudinal studies further demonstrate that SLE patients are more likely to develop frailty phenotypes throughout the entire adult life course, leading researchers to propose that SLE may trigger frailty trajectories associated with aging at an earlier stage, thereby representing a systemic manifestation of accelerated immunosenescence in this population (40, 41).

Beyond physical frailty, cognitive impairment may represent another aging-related systemic phenotype in SLE. Cognitive dysfunction is a recognized manifestation within the neuropsychiatric spectrum of SLE and may arise from chronic inflammation, vascular injury, blood-brain barrier disruption, autoantibody-mediated neuronal injury, and altered T-cell regulation (42). A recent study specifically examined immunosenescence and exhaustion markers in CD4^+^ and CD8^+^ T-cell subsets from patients with SLE and cognitive impairment (43). Compared with SLE patients without cognitive impairment and healthy controls, patients with SLE-related cognitive impairment showed an inverted CD4^+^/CD8^+^ ratio, increased p16^INK4a expression, and altered expression of exhaustion-related co-inhibitory receptors, suggesting that T-cell immunosenescence and exhaustion may contribute to cognitive dysfunction in SLE. However, because the available evidence remains cross-sectional, cognitive impairment should be interpreted as an emerging clinical correlate of accelerated immunosenescence rather than a validated immune-aging endpoint.

#### 2.1.3 Susceptibility to infection and risk of severe infection

Patients with SLE exhibit a significantly higher incidence of severe infections-such as pneumonia, sepsis, and opportunistic infections-compared to the general population. These infections represent a major cause of hospitalization and mortality, a finding consistently validated across multiple center retrospective cohort studies (44, 45). This susceptibility to infection is not only associated with immunosuppressive therapy but also reflects age-mismatched immune dysfunction characteristic of immunosenescence, involving a synergistic effect of chronic inflammation (inflammaging) and age-related immune deficiencies, including remodeling of the innate immune system, complement depletion, and compromised mucosal barriers (10, 46–48). Such infection-prone phenotypes closely mirror those observed in elderly populations and therefore constitute another systemic manifestation of accelerated immunosenescence in SLE.

#### 2.1.4 Impaired vaccine responses

Early studies on pneumococcal vaccines found that SLE patients had significantly lower antibody titers at 1 month and 1 year after vaccination compared with healthy controls, and this blunted response was not fully explained by medication dosage (49, 50). Similarly, SLE patients showed lower seroconversion rates and antibody titers to seasonal influenza vaccines than healthy individuals (51). Reduced vaccine responsiveness is a hallmark feature of immune aging and immunosenescence (12, 52). Multicenter cohorts of COVID-19 vaccination further revealed that, although most SLE patients can generate protective antibody and T-cell responses, overall titers and response rates are still reduced compared with healthy controls (6). Potent immunosuppressive regimens-particularly B-cell depleting agents and myelotoxic drugs-markedly attenuate vaccine immunogenicity, often necessitating additional doses to achieve comparable protection (53). These findings indicate an impaired capacity for immune memory generation and immune reserve, consistent with age-associated immune decline.

Taken together, although SLE primarily affects young adults, these patients exhibit disease patterns closely resembling those of the elderly with respect to cardiovascular events, frailty, cognitive impairment, severe infections, and reduced vaccine responsiveness. This strongly suggests the presence of prominent, system-level accelerated immunosenescence in SLE, linking clinical vaccine hyporesponsiveness to broader immune aging phenotypes (**Table 1**).

**Table 1:** System-level clinical evidence consistent with accelerated immunosenescence in SLE.

Domain	Typical readouts/indices	Clinical meaning	Practical notes/confounders to report
Premature atheroscler osis/CVD	Coronary plaque/calcification on CT; carotid IMT; MI/stroke events; risk scores	Early CVD burden and excess CVD events in SLE	Traditional risks, APS, renal disease, steroid exposure, disease duration
Frailty/vulnerability	Frailty Index (deficit accumulation); gait speed/grip strength (if available); disability/HRQoL	Higher vulnerability → hospitalization/mortality/poor QoL	Age, socioeconomic factors, depression/fatigue, cumulative damage, GC
Cognitive impairment/neuropsychiatric phenotype	Standardized neurocognitive testing; CD4+/CD8+ ratio; p16^INK4a expression; T-cell exhaustion/senescence markers	Emerging systemic phenotype potentially linked to immune aging and T-cell exhaustion in SLE	Evidence remains cross-sectional; consider vascular injury, depression, fatigue, medications, disease activity, and neuropsychiatric SLE heterogeneity
Severe infection susceptibility	Serious infection incidence; infectionrelated Hospitalization/ICU; opportunistic infections	Major hard-outcome driver; informs prophylaxis/vaccine strategy	GC dose, lymphopenia, hypocomplemen temia, prior infections, biologics
Impaired vaccine responses	Seroconversion/seropr otection; antibody titers at 1 mo/6–12 mo; cellular response	“Protection gap” → boosters/timing optimization	B-cell depletion timing, MMF/GC, disease activity,

### 2.2 Cellular level

At the cellular level, accelerated immunosenescence in SLE can be conceptualized as a coordinated remodeling pattern rather than a set of isolated abnormalities in individual cell types. This pattern includes three partially overlapping processes: loss of immune renewal capacity, expansion of age-associated effector populations, and persistence of inflammatory or cytotoxic immune programs. At the lymphoid level, these changes are reflected by contraction of naïve T-cell pools, reduced thymic-output markers, accumulation of terminally differentiated or senescent-like T cells, and expansion of age-associated B-cell and DN2 B-cell populations. At the innate and stromal levels, altered NK-cell function, senescent-like myeloid phenotypes, and BMSC senescence may further reinforce inflammatory tissue damage (54).

#### 2.2.1 T cells

In healthy individuals, the overall composition of T-cell subsets maintains a relatively stable equilibrium following differentiation, activation, and the formation of memory cells (55). However, in SLE, chronic antigenic stimulation coupled with sustained IFN signaling drives CD8^+^ and CD4^+^ T cells prematurely along a trajectory of terminal differentiation-senescence-replicative senescence (56).

Peripheral blood of SLE patients (particularly younger individuals) exhibits significant expansion of multiple terminally differentiated T cells, including CD4^+^KLRG1^+^ and CD8^+^CD57^+^/KLRG1^+^ cells. CD57 and KLRG1, as classic markers of late T-cell differentiation and immune senescence, progressively increase with age in healthy individuals. However, in SLE, they show premature surges, with levels sometimes exceeding those observed in healthy elderly (56, 57).

These senescent-like T cells exhibit features of impaired proliferative reserve and replicative aging, including contraction of the naïve T-cell compartment, reduced thymic output reflected by decreased T-cell receptor excision circles (TRECs) and recent thymic emigrants (RTEs), and expansion of terminally differentiated effector-memory populations (58). Functionally, these cells are not inert; rather, they may retain proinflammatory and cytotoxic activity, including production of IFN-γ and TNF-α, thereby contributing to tissue inflammation and immune-mediated organ damage (56). In SLE, such T-cell senescence-related phenotypes have been associated with disease activity, accumulated damage, lupus nephritis, vascular injury, and, more recently, cognitive impairment, although causal relationships remain incompletely established (43, 56, 59).

A representative example is the vascular-related angiogenic T cell subset. In SLE, the CD4^+^CD28^−^ subsets is significantly expanded and exhibits senescent features including p16 upregulation, telomere shortening, and downregulation of CD27 and CCR7. Its frequency correlates independently with endothelial dysfunction and cardiovascular risk (59).

#### 2.2.2 B cells

B-cell aging in SLE is characterized by a striking shift in lineage distribution, particularly the expansion of age-associated B cells (ABCs) and double-negative 2 (DN2) B cells. These subsets are commonly seen after infection or in the elderly, but in SLE they accumulate to a pathological extent.

ABCs are a T-bet^+^CD11c^+^CD21^−^ B-cell subset that accumulates in older individuals (60). They are highly responsive to Toll-like receptor (TLR7/9) signaling, can autonomously produce autoantibodies, and contribute to renal damage. In SLE, such ABC/ABC-like cells are markedly expanded and are considered an important source of pathogenic autoantibodies.

In human SLE peripheral blood, DN2 B cells (CD19^+^IgD^−^CD27^−^CXCR5^−^T-bet^+^) are enriched and are regarded as functional counterparts of ABCs. The DN2 subset (IgD^−^CD27^−^CD21^−^CD11c^+^T-bet^+^) may even constitute the dominant B-cell population in circulation (61). Expansion of DN2 cells correlates strongly with disease activity and anti-dsDNA antibody titers and is most prominent in young patients, directly reflecting premature aging of the B-cell compartment and its contribution to disease progression through autoantibody production (62).

#### 2.2.3 Other cell populations

Beyond T and B cells, NK cells, monocytes/macrophages, neutrophils, and bone marrow mesenchymal stem cells (BMSCs) in SLE also display age-related transcriptional features.

Total peripheral NK-cell counts are often reduced in SLE, and residual NK cells exhibit a coexistence of functional hyperactivation and exhaustion (63). On the one hand, they overproduce IFN-γ and express activating receptors; on the other, their cytotoxic capacity is significantly impaired. These cells show reduced killer immunoglobulin like receptor (KIR) diversity and expansion of NKG2C^+^ subsets, reminiscent of adaptive NK cells described in the elderly (54, 64).

Small experimental studies have reported that subsets of monocytes in SLE exhibit classical cellular senescence markers, such as p16 upregulation and increased senescence-associated β-galactosidase (SA-β-gal) activity. SLE peripheral blood also contains an increased proportion of senescent-like neutrophils characterized by a CXCR4^hiCD62L^lo phenotype, which correlate positively with disease activity and autoantibody levels (65).

**Figure 2:**
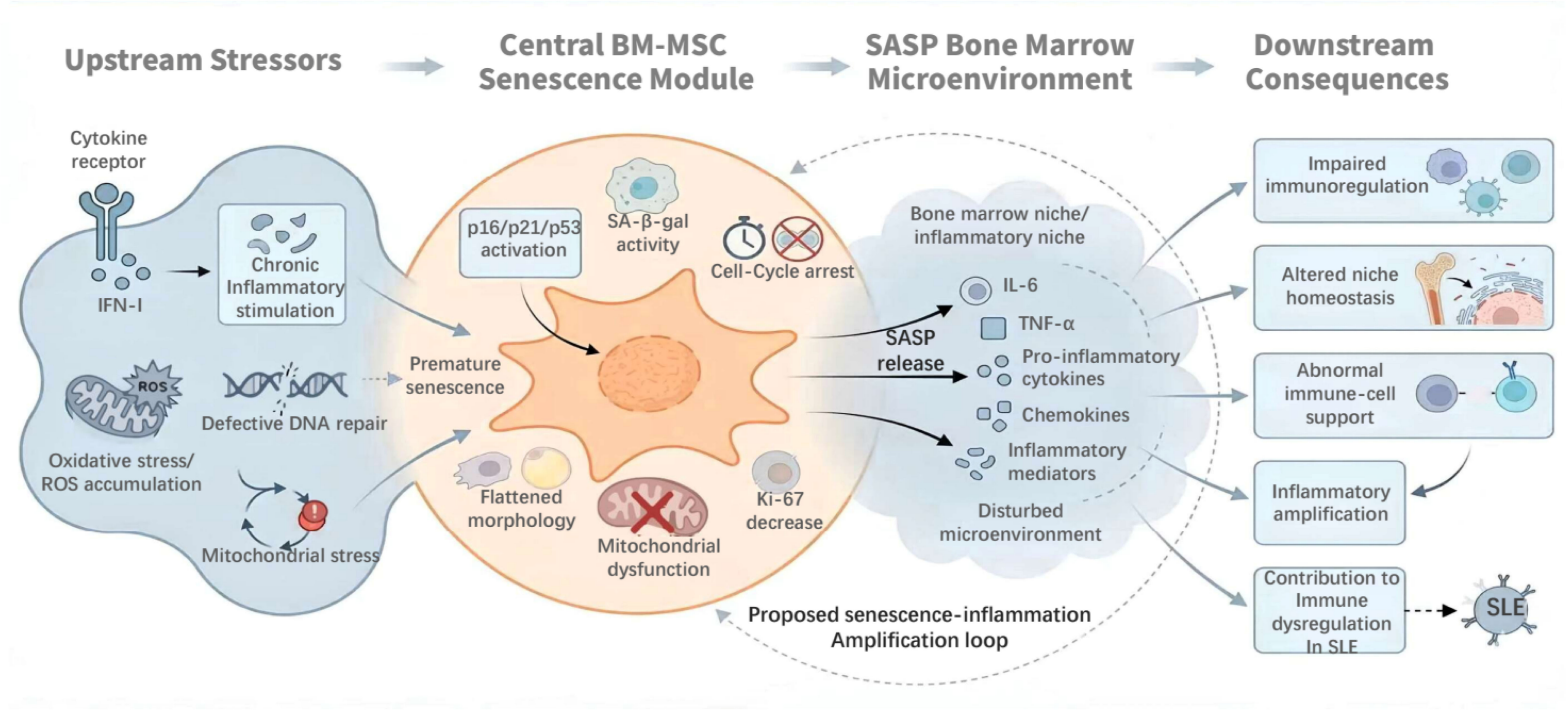
Mechanism of SLE BM-MSC senescence and the pro-inflammatory feedback loop. BM-MSC senescence is depicted as a stromal mechanism through which inflammatory, oxidative and genotoxic stress may be translated into defective marrow immunoregulation in SLE. Acquisition of a senescence-associated state, together with secretory remodelling of the niche, is proposed to impair local homeostasis and reinforce inflammatory signalling, thereby contributing to downstream immune dysregulation. This model is intended to emphasize BM-MSC senescence as a plausible amplifier of disease-associated immune ageing rather than as a singular or definitively proven pathogenic axis.

The most comprehensively studied cellular senescence model in SLE involves BMSCs (66). Compared with age-matched controls, SLE-derived BMSCs show decreased proliferative capacity and a flattened, enlarged morphology (**Figure 2**). They exhibit increased reactive oxygen species (ROS) production, impaired DNA damage repair, activation of p16^INK4a and p53/p21 pathways, and elevated expression of SA-β-gal. These phenotypes closely mirror those of BMSCs from elderly donors. Myeloid-derived suppressor cells (MDSCs) are markedly expanded in SLE and show immunosuppressive properties alongside a senescence-associated secretory phenotype (SASP), correlating with disease activity and organ damage (67, 68).

Across multiple immune cell lineages-including T cells, B cells, NK cells, and myeloid cells-SLE is characterized by the premature expansion and functional remodeling of elderly-associated subsets, together forming a convergent but still incompletely standardized cellular-level picture of accelerated immunosenescence.

However, these findings should be interpreted with caution because definitions of senescent or exhausted immune cells vary substantially across studies. Differences in gating strategies, marker combinations, sample size, disease activity, treatment exposure, infection history, and age matching may contribute to inconsistent results. Therefore, the cellular model proposed here should be viewed as a synthesis of convergent patterns rather than a standardized diagnostic framework.

### 2.3 Molecular level

At the molecular level, SLE exhibits two major dimensions of aging-related molecular alterations. The first is persistent dysregulation of inflammatory mediators resembling inflammaging in older adults. The second is quantifiable acceleration of biological aging reflected by epigenetic and immune clocks, as well as telomere attrition, indicating accelerated biological aging.

#### 2.3.1 Inflammaging-like cytokine profiles

SLE patients display chronically elevated levels of IL-6, IL-10, TNF-α, IL-17, BAFF/BLyS, and type I interferons in peripheral blood, forming a long-standing, low-grade but widespread pro-inflammatory network (69, 70). Several of these mediators correlate directly with organ damage indices and cardiovascular risk (71). Recent reviews have emphasized that abnormal activation of TLR7/9 and nucleic acid-immune complexes co-drive type I interferons and a broad array of inflammatory cytokines, making SLE one of the prototypical human models of inflammaging (72).

#### 2.3.2 Cellular senescence pathways and senescence-associated secretory phenotype features

Accumulating evidence indicates that cells derived from patients with SLE exhibit canonical features of cellular senescence, including increased DNA damage, telomere attrition, and activation of the p53–p21 and p16–Rb signaling pathways (73, 74). These molecular alterations suggest that accelerated cellular senescence may contribute to the high burden of comorbidities observed in SLE (75).

In target organs such as the central nervous system and skin, both animal models and patient tissues demonstrate upregulation of senescence-associated genes and senescence-associated secretory phenotype (SASP) mediators, including IL-6, matrix metalloproteinases, and chemokines (76). The expression of these senescence markers correlates with the severity of neuropsychiatric lupus and cutaneous lesions, and senolytic interventions have been shown to partially reverse pathological changes in experimental models, supporting a functional role of cellular senescence in organ damage associated with SLE (77).

#### 2.3.3 Advancement of DNA methylation clocks (DNAmAge)

DNA methylation-based epigenetic aging analyses provide quantitative tools for assessing accelerated immunosenescence in SLE (78–80). Although some small studies using the classical Horvath epigenetic clock did not detect significant differences (81), a large multiethnic cohort including 323 SLE patients and 99 controls, assessed with the DNAm PhenoAge clock, showed that the epigenetic age of SLE patients exceeded their chronological age by an average of 6.3 years (β_SLE = 6.3, p ≈ 1.8 × 10^−14^). This age acceleration was significantly associated with anti-dsDNA positivity, lupus nephritis, and overall disease severity, providing direct molecular evidence for accelerated biological aging in SLE.

#### 2.3.4 Telomere shortening and reduced telomerase activity

Telomere shortening is another classic hallmark of biological aging. Multiple observational studies and meta-analyses have confirmed that telomere length in peripheral blood mononuclear cells (PBMCs), leukocytes, and CD4^+^ T cells from SLE patients is significantly shorter than in healthy controls (82). This difference is not fully explained by disease activity or cell proliferation, pointing to accelerated baseline biological aging. Telomere shortening correlates positively with disease activity, oxidative stress levels, and antinuclear antibody titers.

In a meta-analysis by Lee et al. that included eight studies with 472 SLE patients and 365 controls, the standardized mean difference (SMD) in telomere length between SLE and control groups was -0.84 (p ≈ 3.3 × 10^−4^), with consistent results across populations, sample types, and detection methods (74). Some studies have found the shortest telomeres within the CD8^+^CD28^−^ memory T-cell subset, indicating long-term antigendriven replicative exhaustion in this population (83).

Concurrently, telomerase (hTERT) expression is reduced, potentially linked to imbalances in mTOR/AMPK signaling and persistent activation of DNA damage response (DDR) pathways (84–86). Mechanistic studies further revealed that CD4^+^ T cells from SLE patients have defective MRE11A nuclease activity in DNA break repair, exacerbating telomeric damage. These telomeric abnormalities were not reversed by methotrexate (MTX) or prednisone treatment, suggesting that telomere aging in SLE is not merely a passive consequence of time but rather reflects inflammation- and metabolism-driven replicative senescence.

At the molecular level, SLE is characterized by an inflammaging-like inflammatory profile dominated by IL-6, TNF-α, BAFF, and type I interferons, together with epigenetic, transcriptomic, and immune-composition signatures indicative of accelerated biological aging and immune clock advancement (70, 87). Collectively, these alterations provide quantifiable molecular evidence consistent with accelerated immunosenescence in SLE. However, telomere length, DNAm clocks, and composite immune-age measures should currently be interpreted as research biomarkers rather than validated clinical tools in SLE, because their prospective predictive value for flares, organ damage, infection, cardiovascular events, and treatment response remains insufficiently established.

Taken together, converging evidence across systemic, cellular, and molecular levels is consistent with that SLE is characterized by a pathological pattern of accelerated immunosenescence. Systemically, young patients accumulate age-related comorbidities prematurely; at the cellular level, T-cell terminal differentiation, naïve T-cell pool contraction, abnormal expansion of ABCs/DN2 B cells (88), and classical senescence phenotypes in BMSCs resemble immune remodeling seen in older adults; at the molecular level, global telomere shortening and markedly accelerated epigenetic aging are evident. These changes largely mirror immunological features of natural aging. They not only delineate the core characteristics of accelerated immunosenescence in SLE but also lay a solid foundation for subsequent work on quantitative assessment, clinical correlations, and interventional targeting of immunosenescence in this disease.

## 3 Clinical translation of immunosenescence

### 3.1 Methods for measuring accelerated immunosenescence

Currently, there is no unified immunosenescence scoring system specific to SLE (89). However, multi-level indicators have been shown to effectively capture immune aging, spanning systemic inflammatory burden, immune cell phenotypes and functions, molecular biological clocks, and tissue-level cellular senescence (12). Integrating these dimensions may enable the development of a standardized quantitative framework suitable for SLE.

Systemic inflammatory burden can be assessed by quantifying chronic inflammatory mediators characteristic of inflammaging, such as IL-6, TNF-α, and CRP using ELISA, multiplex Luminex assays, or commercial inflammatory panels, combined with immune risk profile (IRP) analyses (e.g., inverted CD4/CD8 ratio, abnormal expansion of memory T cells, and chronic viral infection status) (14, 90–93).

Flow cytometry remains the core tool for evaluating immune cell phenotypes (9, 91). T cell immunosenescence can be captured by terminal differentiation/exhaustion markers (CD28^−^, CD57^+^, KLRG1^+^, expansion of TEMRA subsets), loss of TCR diversity, and Treg dysfunction, allowing construction of T-cell immunosenescence scores. Multicolor flow cytometry can also quantify expansions of age-associated B cells (ABCs), double-negative (DN) B cells, and other relevant B-cell subsets, thereby characterizing peripheral immune cell phenotypes and functions (94, 95).

At the molecular level, telomere length in immune cells can be measured using qPCR or flow-FISH as a surrogate for biological age (96, 97). DNA methylation-based epigenetic clocks (e.g., Horvath’s clock) can be used to assess epigenetic age acceleration, while integrated immune-based and inflammatory clocks such as IMM-AGE and iAge incorporate immune cell frequencies, function, and inflammatory proteomics and have shown prognostic relevance in general aging cohorts, but their SLE-specific calibration and clinical validation remain limited (17, 98) (**Table 2**).

**Table 2:** Immunosenescence measurement tools and their validation status in SLE.

Measure	Specimen	Assay/platform	Output	How to interpret clinically	Key caveats	Validation status in SLE
Telomere length	PBMC/leukocytes/ sorted subsets	qPCR (T/S ratio); flow-FISH	TL estimate; “short TL”	Proxy for replicative history/immune reserve	qPCR highly sensitive to preanalytic/plate effects; flow-FISH more reproducible in some clinical settings	Reported in multiple SLE observational studies/meta-analyses; prospective clinical utility remains unclear
DNAm clocks (PhenoAge)	Whole blood DNA/PBMC DNA	DNAm array + PhenoAge algorithm	AgeAccelP heno (Δyears)	Quantifies biological age deviation; can be used as stratifier/endpoint	Strongly affected by cell composition & batch effects; clocks may disagree across cohorts	Limited SLE-specific studies; affected by cell composition and clock choice
Inflammation panels (inflammaging-like burden)	Serum/plasma	ELISA/multiplex cytokines	cytokine levels/composite index	Captures systemic inflammatory load; complements cellular phenotypes	Medication effects; acute infection confounding; diurnal variation	Reported in SLE inflammatory studies; not specific to immunosenescence and confounded by disease activity, infection, and treatment
Integrated immune clock (IMMAGE)	PBMC (deep immunophenoty ping)	High-dim flow/CyTOF + modeling	Immune-age score	Summarizes immune system “state” beyond chronological age	Requires standardization & modeling pipeline; not yet routine clinic	Validated mainly outside SLE; SLE-specific validation lacking
Inflammatory aging clock (iAge)	Plasma inflammatory proteomics	Proteomics + ML model	iAge score	Tracks inflammatory aging; linked to multimorbidit y/frailty/CV aging	Platform dependency; needs reference populations	General aging/inflammatory clock; SLE-specific predictive value unproven
Highlight datum	—	—	—	Reportable highlight: “PhenoAge acceleration ~6.3 years in SLE vs controls” (conference abstract-level evidence)	Label clearly as preliminary/ abstract report	Preliminary illustrative finding only; not a validated SLE threshold or clinical decision tool

Therefore, **Table 2** summarizes not only the biological meaning of each measurement tool but also its current validation status in SLE, distinguishing tools supported by SLE-specific observational data from those extrapolated mainly from general-population aging studies.

### 3.2 Clinical implications of immunosenescence measurement in SLE

**Figure 3:**
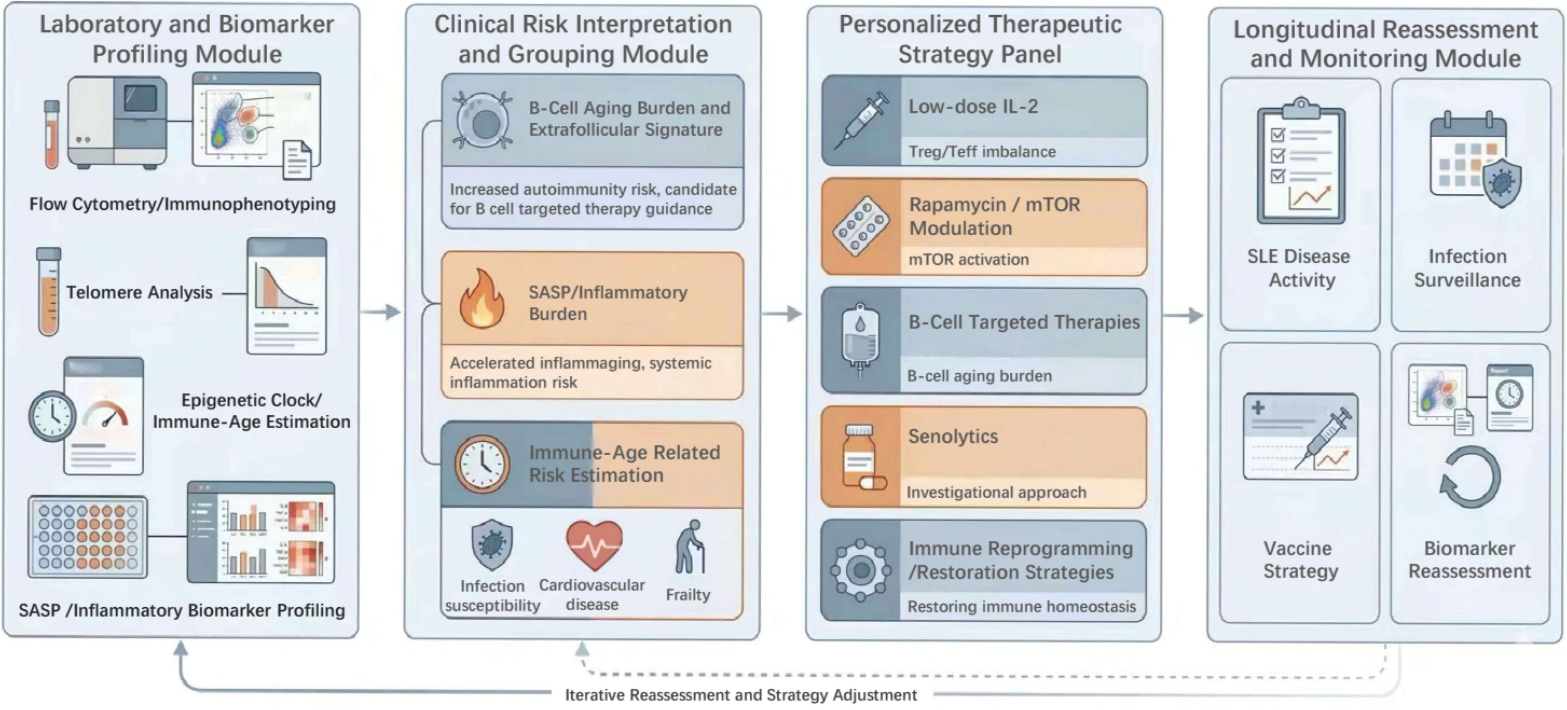
Translational strategy for managing immunosenescence: from biomarker screening to clinical intervention. Immune-age-related readouts are positioned here within an iterative translational framework that links biomarker assessment to risk stratification, individualized intervention and longitudinal reassessment. The central premise is that immunosenescence profiling may help identify vulnerable patients, refine therapeutic selection and improve monitoring of clinically relevant outcomes, including infection susceptibility, cardiometabolic burden and treatment response. The workflow is presented as a strategy for biomarker-guided clinical reasoning in SLE, not as a validated algorithm or standard-of-care pathway.

The clinical application of immunosenescence measurement in SLE should currently be regarded as a translational framework rather than an established standard of care. Existing evidence supports several potential applications, including identification of high-risk patients, refinement of immunosuppressive strategies, optimization of B-cell-targeted therapy, and infection-prevention or vaccination planning. However, prospective studies directly validating immune-age measures as clinical decision tools in SLE are still lacking. Therefore, the following sections distinguish established clinical observations from early mechanistic evidence and more speculative therapeutic applications (14, 17, 99) (**Figure 3**).

#### 3.2.1 A tool for disease stratification

Immunosenescence should be positioned as complementary to, rather than a replacement for, established and emerging SLE stratification approaches. Current stratification frameworks include disease activity and damage indices, organ-specific phenotypes such as lupus nephritis or neuropsychiatric SLE, autoantibody and complement profiles, interferon signatures, transcriptomic or proteomic clusters, and treatment-response phenotypes (100). Immune-aging assessment may add an additional dimension by capturing immune reserve, senescent-cell burden, and vulnerability to infection, vaccination failure, and treatment toxicity.

Within this broader stratification landscape, viewing SLE through the lens of immunosenescence does not necessarily require new drugs; rather, it may help refine the use of existing and emerging therapies in selected patients (8). Patients with high immunosenescence burdens, those with multiple comorbidities, prior severe infections, and a pronounced SASP profile, are generally less tolerant of chronic glucocorticoid and cytotoxic therapies. This perspective may support earlier consideration of steroid-sparing strategies and targeted therapies when clinically appropriate, while recognizing that treatment selection should remain guided by disease activity, organ involvement, safety profile, and existing SLE management recommendations.

In patients whose immune dysregulation is dominated by Treg deficiency and expansion of Th17/Tfh/Tph cells, low-dose IL-2 or interventions targeting the mTOR pathway can be viewed as attempts at rejuvenating immune regulation rather than merely suppressing immunity (101). For those with prominent extrafollicular/age-associated B-cell and DN2 signatures, B-cell-directed therapies (anti-CD20 antibodies, BAFF inhibitors, and, when necessary, CAR-T approaches or hematopoietic stem cell transplantation) may be interpreted as strategies to reset prematurely aged B-cell compartments (8, 102, 103).

Patients with severe depletion of naïve cell pools, high frequencies of senescent T/B cells, or a strong SASP profile require careful coordination of immunosuppressive regimens and vaccination timing, with personalized vaccine schedules (e.g., booster doses, high-dose formulations, adjuvanted vaccines where permissible) and a lower threshold for prophylactic and early therapeutic anti-infective measures.

When senolytics, mesenchymal stromal cell-based therapies (104), or more precise metabolic modulators become clinically available, individuals with clear evidence of accelerated immunosenescence, such as shortened telomeres, robust SASP profiles, or a high burden of age-associated comorbidities, will likely constitute the most rational initial target population. For example, the BCL-2 inhibitor ABT-263 (navitoclax), a senolytic agent, can deplete senescent T/B cells and reduce SASP cytokines, thereby markedly ameliorating lupus-like disease in MRL/lpr mice.

#### 3.2.2 Guiding and monitoring immune-reprogramming therapies

Immunosenescence markers also represent potential therapeutic targets and pharmacodynamic endpoints for immune-reprogramming interventions in SLE (17). Several illustrative examples have already emerged. These examples should be interpreted as early translational or mechanistic evidence rather than established anti-immunosenescence therapies. Their relevance to immunosenescence lies mainly in their potential to modulate immune regulation, restore immune repertoire diversity, or reset pathogenic immune compartments, rather than in proven clinical reversal of immune aging in SLE.

Low-dose IL-2 has shown the ability to selectively expand regulatory T cells and rebalance T-cell subsets in SLE, providing a mechanistic rationale for immune-regulatory restoration (101). However, it should not yet be described as a validated immune-rejuvenation therapy, because available trials have focused mainly on disease activity and immunological readouts rather than prospective immune-age endpoints. SLE is characterized by acquired IL-2 deficiency and Treg dysfunction. Low-dose IL-2 selectively expands Treg cells and is considered an effective means of partially resetting the immune system. Open-label cohorts and randomized controlled trials have shown that low-dose IL-2 can significantly increase Treg counts, reduce the Th17/Treg ratio, and concomitantly lower SLEDAI scores, enabling steroid reduction (101).

Building on this, Zhao and colleagues applied a combination of low-dose IL-2 plus rapamycin in a cohort of refractory SLE patients (105). After 24 weeks, approximately one quarter of patients achieved clinical remission, accompanied by increased absolute Treg numbers and a reduced Th17/Treg ratio. The authors proposed that this regimen represents a strategy for inducing immune tolerance. Rapamycin and related mTOR-modulating strategies provide another mechanistic link between metabolic stress, T-cell dysfunction, and immune remodeling in SLE. Early studies suggest that mTOR inhibition may reduce disease activity and normalize abnormal T-cell activation pathways, but the evidence base remains limited and does not establish rapamycin as a clinically validated anti-immunosenescence intervention (84).

Autologous hematopoietic stem cell transplantation (HSCT) offers a unique window into immune system regeneration. In severe refractory SLE, HSCT has been used to ablate and rebuild the immune system (106). Follow-up 6–12 months after HSCT has revealed restoration of polyclonal TCR diversity, correction of abnormal immune gene expression, and broader changes in T cells, B cells, plasmablasts, and NK cells (107). Increased Treg levels following HSCT are a common observation, and subsequent clinical improvements have been linked to a marked reset of Treg TCR diversity. However, HSCT is associated with substantial treatment-related risks and remains unsuitable for most SLE patients, though it provides compelling proof-of-principle that advanced autoimmune immunosenescence can be partially reversed. Nevertheless, HSCT is applicable only to highly selected patients with severe refractory disease and carries substantial treatment burden and safety concerns. Its value in the context of immunosenescence is therefore best interpreted as proof-of-concept for immune-system resetting rather than as a broadly applicable anti-aging strategy in SLE (108).

From a methodological standpoint, multidimensional immune clocks such as IMMAGE and iAge have been validated in the general population as predictors of mortality, cardiovascular events, and frailty (14). In trials targeting immunosenescence, immune age should be included as a key secondary endpoint to assess whether interventions genuinely reverse senescent phenotypes rather than merely transiently suppressing inflammation. No prospective SLE trial has yet implemented such a design, highlighting a key direction for future clinical studies.

#### 3.2.3 Optimization of B-cell-targeted therapy

B cells lie at the core of SLE immunopathology and represent a critical point of convergence between immunosenescence and autoimmunity. ABCs and T-bet^+^ B cells are markedly expanded in the elderly, in chronic infections, and in SLE, and are considered highly pathogenic and treatment-resistant (60). Their characterization has important implications for refining B-cell-targeted therapies.

Cross-omics studies by Ramsköld and colleagues have shown that the BAFF inhibitor belimumab significantly reduces circulating CD20^+^ B cells and plasmablasts while partially restoring B-cell homeostasis (5) (109). Early after subcutaneous belimumab, circulating T-bet^+^ B cells (ABC-like phenotypes) decline, and the magnitude of this reduction correlates strongly with clinical response, suggesting that T-bet^+^ B cells may serve as an early response biomarker for belimumab.

A recent study on IgA2^+^ B cells and IgA2 anti-dsDNA antibodies found that in SLE patients receiving rituximab induction followed by belimumab, belimumab could further clear IgA2 plasmablasts and CD11c^+^T-bet^+^ B cells (110). This indicates that these immunosenescent B-cell populations may persist despite conventional B-cell depletion and may be linked to disease relapse and refractoriness.

In 2022, a small landmark case series in refractory SLE showed that CD19 CAR-T-cell therapy induced deep B-cell depletion, clinical remission, and reconstitution of predominantly naïve, non-class-switched B cells after B-cell recovery (103, 111, 112). These findings provide proof-of-concept that pathogenic B-cell compartments can be reset in selected patients. However, the evidence remains based on small, highly selected cohorts with limited long-term follow-up, and larger controlled studies are needed to define durability, safety, patient selection, and cost-effectiveness.

#### 3.2.4 Infection prevention and vaccination strategies

In immunosenescent populations, infection risk is substantially elevated while vaccine immunogenicity is clearly reduced, exemplified by increased susceptibility and weaker vaccine responses to pneumonia, herpes zoster, and COVID-19 (48, 113).

In SLE, although no large-scale cohort has yet systematically used immunosenescence scores to guide vaccination strategies, existing evidence is sufficient to support that SLE patients with pronounced immunosenescence (e.g., marked T/B-cell senescent phenotypes and high inflammatory clock scores) constitute an inherently vulnerable group for infections (114). When such patients receive intense immunosuppression (cyclophosphamide, myelotoxic agents, or combinations of multiple biologics), they require stricter prophylactic measures (e.g., vaccination, *Pneumocystis jirovecii* pneumonia prophylaxis, herpes zoster vaccination) and more carefully calibrated immunosuppressive intensity.

For SLE patients with recurrent infections, prolonged exposure to aggressive immunosuppression, or advanced age, immunosenescence evaluation may help determine optimal vaccination windows, booster frequency, and opportunities for tapering immunosuppression. In the future, if high-immunosenescence subgroups can be reproducibly identified, immune-aging assessment may help inform steroid-sparing strategies, vaccination timing, infection prophylaxis, and treatment-intensity decisions, but such applications require prospective validation.

### 3.3 Evidence hierarchy and translational boundaries

The translational implications of immunosenescence in SLE should be interpreted according to the maturity of evidence. Established clinical observations include the excess burden of cardiovascular disease, severe infection, frailty, cognitive impairment, and impaired vaccine responses in SLE (4, 24–28). Early or mechanistic evidence includes the expansion of senescent-like T cells, ABC/DN2 B cells, telomere shortening, epigenetic age acceleration, and inflammatory-clock abnormalities. More speculative applications include the use of immune clocks for clinical decision-making, selection of immune-rejuvenation therapies, senolytic approaches, and cellular immune-resetting strategies.

Accordingly, low-dose IL-2, rapamycin, HSCT, and CAR-T therapy should not be framed as established anti-immunosenescence treatments in SLE (101, 115). Low-dose IL-2 and rapamycin provide mechanistic evidence for immune-regulatory or metabolic modulation, whereas HSCT and CAR-T therapy provide proof-of-concept evidence for immune-system resetting in selected patients with severe or refractory disease. However, these approaches remain limited by sample size, patient selection, safety concerns, treatment burden, cost, and incomplete randomized controlled evidence.

## 4 Limitations and unanswered questions

The current evidence base for accelerated immunosenescence in SLE is substantial but uneven. Most studies are cross-sectional, retrospective, or based on relatively small cohorts, making it difficult to determine whether immune aging is a causal driver of SLE pathogenesis, a consequence of chronic inflammation and treatment exposure, or a parallel process shaped by both disease biology and accumulated damage. Longitudinal studies with repeated immune profiling are needed to clarify temporal relationships among immune-aging markers, disease activity, organ damage, infection risk, and treatment response (81, 116).

Various immune-aging markers, including terminally differentiated T cells, ABC/DN2-like B cells, telomere length, DNAm clocks, and SASP-related signatures, are influenced by age, ancestry, disease phenotype, infection history, comorbidities, and medication exposure (117). These confounders limit cross-cohort comparability and may explain why immune-aging signals vary across studies (60).

It should also be emphasized that immune clocks, telomere length, and other quantitative immune-aging measures remain insufficiently validated as prospective biomarkers in SLE (118). Future studies should evaluate whether these markers predict clinically meaningful outcomes, including flares, damage accrual, severe infection, cardiovascular events, vaccine response, and treatment toxicity, beyond established clinical predictors.

Similarly, immune-aging markers such as telomere length, DNA methylation clocks, inflammatory clocks, and composite immune-age scores should currently be considered research tools rather than validated clinical biomarkers in SLE. Although some markers have shown associations with disease severity or immune dysfunction, their prospective predictive value for flares, damage accrual, infection, cardiovascular events, and treatment response remains insufficiently established in SLE-specific cohorts.

Finally, inconsistent definitions and gating criteria for key cell populations (e.g., ABCs)-coupled with their significant functional heterogeneity-undermine inter-study consistency and impede standardized application, compromising the reliability of findings across research settings.

## 5 Conclusions and perspectives

The immunosenescence perspective provides a useful conceptual framework for interpreting selected clinical and immunological features of SLE. It encourages clinicians to move beyond the traditional two dimensional framework of disease activity-organ damage and to incorporate a third dimension of immune reserve-immune exhaustion/senescence when interpreting early multimorbidity, recurrent infections, frailty, and heightened sensitivity to treatment toxicity in young patients (89, 119).

This perspective suggests that marked depletion of naïve T cells, expansion of CD28^−^/CD57^+^ T cells or age-associated/DN2 B-cell subsets, and persistent type I interferon and SASP signatures may be considered early warning signs of declining immune reserve-even if they are not yet formal biomarkers (19, 120). At the same time, these features provide theoretical support for clinical strategies such as prioritizing steroid-sparing regimens, tailoring infection prophylaxis and vaccination plans, and selectively applying rebalancing or resetting interventions in patients whose immune age exceeds their chronological age.

Immunosenescence-oriented thinking also clarifies priorities for prospective research.

Mechanistic studies are needed to elucidate key nodes within type I interferon signaling, mTOR/AMPK pathways, mitochondrial quality control, DNA damage response, and SASP regulation, and to identify targets that can meaningfully alter immunosenescence trajectories within acceptable toxicity (19, 72, 84, 104, 110). Longitudinal multi-omics studies across diverse populations and disease phenotypes are required to identify individuals at high risk of rapid immunosenescence progression, to determine critical windows in the disease course when immunosenescence becomes fixed, and to define interventions capable of effectively reversing these trajectories. If such efforts are successful, SLE will not only benefit from advances in aging research but may also become a central testbed for strategies aimed at preserving and restoring youthful immune states in chronic inflammatory diseases.

Current evidence supports immunosenescence as a biologically plausible and clinically informative lens through which to interpret selected features of SLE, including premature multimorbidity, infection vulnerability, vaccine hyporesponsiveness, cellular immune remodeling, and molecular aging signatures. However, its causal role, prognostic value, and therapeutic manipulability remain insufficiently established.

Importantly, immunosenescence should not be equated with inflammaging, treatment-related toxicity, or accumulated damage. Inflammaging represents a chronic inflammatory milieu that may drive or amplify immune aging, whereas treatment toxicity and accumulated damage reflect downstream clinical consequences of therapy, disease activity, comorbidities, and irreversible organ injury. Immunosenescence, by contrast, refers to remodeling of immune architecture and function, including reduced immune renewal, loss of naïve-cell reserve, restricted immune-repertoire diversity, and expansion of terminally differentiated or senescent immune subsets. Recognizing these distinctions is essential for using immune aging as a complementary framework rather than a replacement for established clinical models of SLE (23).

Future longitudinal and interventional studies should determine whether immune-aging measures provide incremental predictive value beyond conventional indices of disease activity, organ damage, interferon signatures, organ-specific phenotypes, and molecular endotypes. Until such evidence is available, immunosenescence should be regarded as a promising conceptual and translational framework, not as a validated clinical stratification system (100).

## References

[1] Dai X Fan Y Zhao X . Systemic lupus erythematosus: updated insights on the pathogenesis, diagnosis, prevention and therapeutics. Signal Transduction Targeted Ther. (2025) 10:102. doi: 10.1038/s41392-025-02168-0 40097390 PMC11914703

[2] Tian J Zhang D Yao X Huang Y Lu Q . Global epidemiology of systemic lupus erythematosus: a comprehensive systematic analysis and modelling study. Ann Rheumatic Dis. (2023) 82:351–6. doi: 10.1136/ard-2022-223035 36241363 PMC9933169

[3] Muñoz-Grajales C Yilmaz EB Svenungsson E Touma Z . Systemic lupus erythematosus and damage: what has changed over the past 20 years? Best Pract Research: Clin Rheumatol. (2023) 37:101893. doi: 10.1016/j.berh.2023.101893 37993371

[4] Pego-Reigosa JM Nicholson L Pooley N Langham S Embleton N Marjenberg Z . The risk of infections in adult patients with systemic lupus erythematosus: systematic review and meta-analysis. Rheumatology. (2021) 60:60–72. doi: 10.1093/rheumatology/keaa478 33099651 PMC7785308

[5] Rodziewicz M Dyball S Lunt M McDonald S Eutton S Parker B . Early infection risk in patients with systemic lupus erythematosus treated with rituximab or belimumab from the British Isles Lupus Assessment Group Biologics Register (BILAG-BR): a prospective longitudinal study. Lancet Rheumatol. (2023) 5:e284–92. doi: 10.1016/S2665-9913(23)00091-7 38251591

[6] Petri M Joyce D Haag K Fava A Goldman DW Zhong D . Effect of systemic lupus erythematosus and immunosuppressive agents on COVID-19 vaccination antibody response. Arthritis Care Res. (2023) 75:1878–85. doi: 10.1002/acr.25094 36714913 PMC10387122

[7] Chatterjee R Kommaraju SY Mettingal Ramakrishnan S Ravikumar KL Aggarwal A . Immunogenicity, safety and adverse events of sequential vaccination with a 10-valent pneumococcal conjugate vaccine (PCV10) and PPSV23 compared with PPSV23 alone in systemic lupus erythematosus. Lupus Sci Med. (2025) 12:e001551. doi: 10.1136/lupus-2025-001551 40921623 PMC12421157

[8] Drosos GC Vedder D Houben E Boekel L Atzeni F Badreh S . EULAR recommendations for cardiovascular risk management in rheumatic and musculoskeletal diseases, including systemic lupus erythematosus and antiphospholipid syndrome. Ann Rheumatic Dis. (2022) 81:768–79. doi: 10.1136/annrheumdis-2021-221733 35110331

[9] Jiang J Yang M Zhu H Long D He Z Liu J . CD4^+^ CD57^+^ senescent T cells as promoters of systemic lupus erythematosus pathogenesis and the therapeutic potential of senolytic BCL-2 inhibitor. Eur J Immunol. (2024) 54:2350603. doi: 10.1002/eji.202350603 38752316

[10] Mittal R Saavedra D Mittal M Lemos JR Hirani K . Inflammasome activation and accelerated immune aging in autoimmune disorders. Front Aging. (2025) 6:1688060. doi: 10.3389/fragi.2025.1688060 41089406 PMC12517589

[11] Liu Z Liang Q Ren Y Guo C Ge X Wang L . Immunosenescence: molecular mechanisms and diseases. Signal Transduction Targeted Ther. (2023) 8:200. doi: 10.1038/s41392-023-01451-2 37179335 PMC10182360

[12] Fu Y Wang B Alu A Hong W Lei H He X . Immunosenescence: signaling pathways, diseases and therapeutic targets. Signal Transduction Targeted Ther. (2025) 10:250. doi: 10.1038/s41392-025-02371-z 40769978 PMC12328749

[13] Effros RB . Roy walford and the immunologic theory of aging. Immun Ageing. (2005) 2:7. doi: 10.1186/1742-4933-2-7 15850487 PMC1131916

[14] Sayed N Huang Y Nguyen K Krejciova-Rajaniemi Z Grawe AP Gao T . An inflammatory aging clock (iAge) based on deep learning tracks multimorbidity, immunosenescence, frailty and cardiovascular aging. Nat Aging. (2021) 1:598–615. doi: 10.1038/s43587-021-00082-y 34888528 PMC8654267

[15] Montoya-Ortiz G . Immunosenescence, aging, and systemic lupus erythematous. Autoimmune Dis. (2013) 2013:1–15. doi: 10.1155/2013/267078 24260712 PMC3821895

[16] Kalim H Wahono C Permana B Pratama M Handono K . Association between senescence of T cells and disease activity in patients with systemic lupus erythematosus. Rheumatology. (2021) 59:292–301. doi: 10.5114/reum.2021.110318 34819703 PMC8609380

[17] Alpert A Pickman Y Leipold M Rosenberg-Hasson Y Ji X Gaujoux R . A clinically meaningful metric of immune age derived from high-dimensional longitudinal monitoring. Nat Med. (2019) 25:487–95. doi: 10.1038/s41591-019-0381-y 30842675 PMC6686855

[18] Gong Q Sharma M Glass MC Kuan EL Chander A Singh M . Multi-omic profiling reveals age-related immune dynamics in healthy adults. Nature. (2025) 648:696–706. doi: 10.1038/s41586-025-09686-5 41162704 PMC12711581

[19] Narendra R Van Phan H Patterson SL Almonte-Loya A Lydon EC Lanata C . Epigenetic attenuation of interferon signaling is associated with aging-related improvements in systemic lupus erythematosus. Sci Transl Med. (2025) 17:eadt5550. doi: 10.1126/scitranslmed.adt5550 40561001 PMC13012812

[20] Su QY Zheng XX Han XT Li Q Gao YR Zhang SX . The role of age-associated B cells in systemic lupus erythematosus. J Autoimmun. (2025) 154:103433. doi: 10.1016/j.jaut.2025.103433 40334618

[21] Somers EC Goodrich JM Wang L Harlow SD Marder W Hassett AL . Associations between CD70 methylation of T cell DNA and age in adults with systemic lupus erythematosus and population controls: the Michigan Lupus Epidemiology & Surveillance (MILES) program. J Autoimmun. (2024) 142:103137. doi: 10.1016/j.jaut.2023.103137 38064919 PMC10957300

[22] Min M Egli C Dulai AS Sivamani RK . Critical review of aging clocks and factors that may influence the pace of aging. Front Aging. (2024) 5:1487260. doi: 10.3389/fragi.2024.1487260 39735686 PMC11671503

[23] Santoro A Bientinesi E Monti D . Immunosenescence and inflammaging in the aging process: age-related diseases or longevity? Ageing Res Rev. (2021) 71:101422. doi: 10.1016/j.arr.2021.101422 34391943

[24] Frostegård J . Systemic lupus erythematosus and cardiovascular disease. J Internal Med. (2023) 293:48–62. doi: 10.1111/joim.13557 35982610 PMC10087345

[25] Wang X Yan S Liu C Xu Y Wan L Wang Y . Fracture risk and bone mineral density levels in patients with systemic lupus erythematosus: a systematic review and meta-analysis. Osteoporosis Int. (2016) 27:1413–23. doi: 10.1007/s00198-015-3449-7 26753541

[26] Sang N Zhang H Zhang M Zhang M Zhu Y Chen H . Prevalence of frailty and prefrailty in systemic lupus erythematosus: a systematic review and meta-analysis. Semin Arthritis Rheumatism. (2025) 72:152709. doi: 10.1016/j.semarthrit.2025.152709 40086156

[27] Zhang M Wang Y Wang Y Bai Y Gu D . Association between systemic lupus erythematosus and cancer morbidity and mortality: findings from cohort studies. Front Oncol. (2022) 12:860794. doi: 10.3389/fonc.2022.860794 35600353 PMC9115099

[28] Rua-Figueroa I García De Yébenes MJ Martinez-Barrio J Galindo Izquierdo M Calvo Alén J Fernandez-Nebro A . SLESISR: an improved score for prediction of serious infection in patients with systemic lupus erythematosus based on the RELESSER prospective cohort. Lupus Sci Med. (2024) 11:e001096. doi: 10.1136/lupus-2023-001096 38589223 PMC11015315

[29] Mendoza-Pinto C Munguía-Realpzo P García-Carrasco M Godinez-Bolaños K Rojas-Villarraga A Morales-Etchegaray I . Asymptomatic coronary artery disease assessed by coronary computed tomography in patients with systemic lupus erythematosus: a systematic review and meta-analysis. Eur J Internal Med. (2022) 100:102–9. doi: 10.1016/j.ejim.2022.04.001 35410814

[30] Zinglersen L Zinglersen AH Myhr KA Hermansen ML Kofoed KF Fuchs A . Coronary artery calcification progression and renal involvement in patients with systemic lupus erythematosus: a longitudinal cohort study. Rheumatol Int. (2025) 45:26. doi: 10.1007/s00296-025-05785-8 39804493 PMC11729070

[31] Ajoolabady A Pratico D Lin L Mantzoros CS Bahijri S Tuomilehto J . Inflammation in atherosclerosis: pathophysiology and mechanisms. Cell Death Dis. (2024) 15:817. doi: 10.1038/s41419-024-07166-8 39528464 PMC11555284

[32] Snijckers RPM Foks AC . Adaptive immunity and atherosclerosis: aging at its crossroads. Front Immunol. (2024) 15:1350471. doi: 10.3389/fimmu.2024.1350471 38686373 PMC11056569

[33] Weyand CM Goronzy JJ . Aging of the immune system. Mechanisms and therapeutic targets. Ann Am Thorac Soc. (2016) 13:S422–8. doi: 10.1513/AnnalsATS.201602-095AW 28005419 PMC5291468

[34] Papazoglou N Sfikakis PP Tektonidou MG . Atherosclerotic plaque progression and incident cardiovascular events in a 10-year prospective study of patients with systemic lupus erythematosus: the impact of persistent cardiovascular risk factor target attainment and sustained DORIS remission. Arthritis Rheumatol. (2025) 77:716–26. doi: 10.1002/art.43097 39721769 PMC12123254

[35] Pineau CA Lee C Ramsey-Goldman R Clarke AE Bernatsky S . The second hit: comorbidities in systemic lupus erythematosus. Future Rheumatol. (2007) 2:497–506. doi: 10.2217/17460816.2.5.497

[36] Legge A Kirkland S Rockwood K Andreou P Bae SC Gordon C . Construction of a frailty index as a novel health measure in systemic lupus erythematosus. J Rheumatol. (2020) 47:72–81. doi: 10.3899/jrheum.181338 30988130 PMC6800806

[37] Keramiotou K Anagnostou C Konstantonis G Fragiadaki K Kataxaki E Sfikakis PP . SLICC-frailty index is independently associated with impaired physical function, activities of daily living and quality of life measures. Rheumatology. (2022) 61:3808–13. doi: 10.1093/rheumatology/keac001 35015829

[38] Lieber SB Nahid M Paget S Berman JR Barbhaiya M Sammaritano LR . Evaluation of a patient-reported frailty tool in women with systemic lupus erythematosus. J Rheumatol. (2022) 49:60–7. doi: 10.3899/jrheum.201466 34470795 PMC10279522

[39] Katz PP Andrews J Yazdany J Schmajuk G Trupin L Yelin E . Is frailty a relevant concept in SLE? Lupus Sci Med. (2017) 4:e000186. doi: 10.1136/lupus-2016-000186 28243456 PMC5294024

[40] Singh A Gamboa-Cárdenas RV Pimentel-Quiroz VR Reátegui-Sokolova C Rodriguez-Bellido Z Pastor-Asurza CA . Systemic lupus international collaborating clinics-frailty index predicts hospitalisations in the Almenara lupus cohort. Lupus Sci Med. (2025) 12:e001624. doi: 10.1136/lupus-2025-001624 40659389 PMC12258343

[41] Katz P Dall'Era M Plantinga L Barbour KE Greenlund KJ Yazdany J . Measuring frailty in systemic lupus erythematosus. Arthritis Care Res. (2025) 77:700–9. doi: 10.1002/acr.25479 39648405

[42] Bertsias GK Ioannidis JPA Aringer M Bollen E Bomb . EULAR recommendations for the management of systemic lupus erythematosus with neuropsychiatric manifestations: report of a task force of the EULAR standing committee for clinical affairs. Ann Rheumatic Dis. (2010) 69:2074–82. doi: 10.1136/ard.2010.130476 20724309

[43] Cimé-Aké E Lima G Godínez-Lazarini E Juárez S Marín-López H Flores-Hernández D . Immunosenescent and exhausted T cells in systemic lupus erythematosus patients with cognitive impairment. Clin Immunol. (2026) 282:110635. doi: 10.1016/j.clim.2025.110635 41183629

[44] Zhao K Xie H Li L Esdaile JM Aviña-Zubieta JA . Increased risk of severe infections and mortality in patients with newly diagnosed systemic lupus erythematosus: a population-based study. Rheumatology. (2021) 60:5300–9. doi: 10.1093/rheumatology/keab219 33751035

[45] Dhital R Guma M Poudel DR Chambers C Kalunian K . Infection-related hospitalisation in young adults with systemic lupus erythematosus: data from the national inpatient sample. Lupus Sci Med. (2023) 10:e000851. doi: 10.1136/lupus-2022-000851 37019477 PMC10083864

[46] Karpuzoglu E Holladay SD Gogal RM . Inflammaging: triggers, molecular mechanisms, immunological consequences, sex differences, and cutaneous manifestations. Front Immunol. (2025) 16:1704203. doi: 10.3389/fimmu.2025.1704203 41425557 PMC12714610

[47] Ali AY Zahran SA Eissa M Kashef MT Ali AE . Gut microbiota dysbiosis and associated immune response in systemic lupus erythematosus: impact of disease and treatment. Gut Pathog. (2025) 17:10. doi: 10.1186/s13099-025-00683-7 39966979 PMC11834511

[48] He J Li Z . Dilemma of immunosuppression and infection risk in systemic lupus erythematosus. Rheumatology. (2023) 62:i22–9. doi: 10.1093/rheumatology/keac678 36987605 PMC10050939

[49] Jarrett MP Schiffman G Barland P Grayzel AI . Impaired response to pneumococcal vaccine in systemic lupus erythematosus. Arthritis Rheumatism. (1980) 23:1287–93. doi: 10.1002/art.1780231110 7447963

[50] Grabar S Groh M Bahuaud M Le Guern V Costedoat-Chalumeau N Mathian A . Pneumococcal vaccination in patients with systemic lupus erythematosus: a multicenter placebo-controlled randomized double-blind study. Vaccine. (2017) 35:4877–85. doi: 10.1016/j.vaccine.2017.07.094 28784280

[51] Liao Z Tang H Xu X Liang Y Xiong Y Ni J . Immunogenicity and safety of influenza vaccination in systemic lupus erythematosus patients compared with healthy controls: a meta-analysis. PloS One. (2016) 11:e0147856. doi: 10.1371/journal.pone.0147856 26845680 PMC4742052

[52] Hou Y Chen M Bian Y Hu Y Chuan J Zhong L . Insights into vaccines for elderly individuals: from the impacts of immunosenescence to delivery strategies. NPJ Vaccines. (2024) 9:77. doi: 10.1038/s41541-024-00874-4 38600250 PMC11006855

[53] Mackay M Wagner CA Pinckney A Cohen JA Wallace ZS Khosroshahi A . Prospective SARS-CoV-2 additional vaccination in immunosuppressant-treated individuals with autoimmune diseases in a randomized controlled trial. JCI Insight. (2026) 11:e191266. doi: 10.1172/jci.insight.191266 41289027 PMC12890511

[54] Terekhova M Swain A Bohacova P Aladyeva E Arthur L Laha A . Human immune aging. Immunity. (2025) 58:2646–69. doi: 10.1016/j.immuni.2025.10.009 41175875

[55] Terekhova M Swain A Bohacova P . Single-cell atlas of healthy human blood unveils age-related loss of NKG2C+GZMB-CD8+ memory T cells and accumulation of type 2 memory T cells. Immunity. (2023) 56:2836–2854.e9. doi: 10.1016/j.immuni.2023.10.013 37963457

[56] Xiong H Cui M Kong N Jing J Xu Y Liu X . Cytotoxic CD161-CD8+ TEMRA cells contribute to the pathogenesis of systemic lupus erythematosus. Ebiomedicine. (2023) 90:104507. doi: 10.1016/j.ebiom.2023.104507 36893588 PMC10011749

[57] Guan Y Cao M Wu X Yan J Hao Y Zhang C . CD28null T cells in aging and diseases: From biology to assessment and intervention. Int Immunopharmacol. (2024) 131:111807. doi: 10.1016/j.intimp.2024.111807 38471362

[58] Bohacova P Terekhova M Tsurinov P Mullins R Husarcikova K Shchukina I . Multidimensional profiling of human T cells reveals high CD38 expression, marking recent thymic emigrants and age-related naive T cell remodeling. Immunity. (2024) 57:2362–2379.e10. doi: 10.1016/j.immuni.2024.08.019 39321807 PMC13138122

[59] Tilman G Bouzin C Aydin S Tamirou F Galant C Coulie PG . High p16^INK4a^ , a marker of cellular senescence, is associated with renal injury, impairment and outcome in lupus nephritis. Rmd Open. (2021) 7:e001844. doi: 10.1136/rmdopen-2021-001844 34686545 PMC8543753

[60] Xie G Chen X Gao Y Yang M Zhou S Lu L . Age-associated B cells in autoimmune diseases: Pathogenesis and clinical implications. Clin Rev Allergy Immunol. (2025) 68:18. doi: 10.1007/s12016-025-09021-w 39960645 PMC11832777

[61] Jenks SA Cashman KS Zumaquero E Marigorta UM Patel AV Wang X . Distinct effector B cells induced by unregulated toll-like receptor 7 contribute to pathogenic responses in systemic lupus erythematosus. Immunity. (2018) 49:725–739.e6. doi: 10.1016/j.immuni.2018.08.015 30314758 PMC6217820

[62] Phalke S Rivera-Correa J Jenkins D Flores Castro D Giannopoulou E Pernis AB . Molecular mechanisms controlling age-associated B cells in autoimmunity*. Immunol Rev. (2022) 307:79–100. doi: 10.1111/imr.13068 35102602

[63] Liu M Liang S Zhang C . NK cells in autoimmune diseases: protective or pathogenic? Front Immunol. (2021) 12:624687. doi: 10.3389/fimmu.2021.624687 33777006 PMC7994264

[64] Segerberg F Lundtoft C Reid S Hjorton K Leonard D Nordmark G . Autoantibodies to killer cell immunoglobulin-like receptors in patients with systemic lupus erythematosus induce natural killer cell hyporesponsiveness. Front Immunol. (2019) 10:2164. doi: 10.3389/fimmu.2019.02164 31572377 PMC6749077

[65] Kuga T Chiba A Murayama G Hosomi K Nakagawa T Yahagi Y . Enhanced GATA4 expression in senescent systemic lupus erythematosus monocytes promotes high levels of IFNα production. Front Immunol. (2024) 15:1320444. doi: 10.3389/fimmu.2024.1320444 38605949 PMC11007064

[66] Gao L Bird AK Meednu N Dauenhauer K Liesveld J Anolik J . Bone marrow–derived mesenchymal stem cells from patients with systemic lupus erythematosus have a senescence‐associated secretory phenotype mediated by a mitochondrial antiviral signaling protein–interferon‐β feedback loop. Arthritis Rheumatol. (2017) 69:1623–35. doi: 10.1002/art.40142 28471483 PMC5560120

[67] Gu Z Cao X Jiang J Li L Da Z Liu H . Upregulation of p16INK4A promotes cellular senescence of bone marrow-derived mesenchymal stem cells from systemic lupus erythematosus patients. Cell Signalling. (2012) 24:2307–14. doi: 10.1016/j.cellsig.2012.07.012 22820504

[68] Li D Qi J Wang J Pan Y Li J Xia X . Protective effect of dihydroartemisinin in inhibiting senescence of myeloid-derived suppressor cells from lupus mice via Nrf2/HO-1 pathway. Free Radical Biol Med. (2019) 143:260–74. doi: 10.1016/j.freeradbiomed.2019.08.013 31419476

[69] Tsokos GC . The immunology of systemic lupus erythematosus. Nat Immunol. (2024) 25:1332–43. doi: 10.1038/s41590-024-01898-7 39009839

[70] Crow MK . Pathogenesis of systemic lupus erythematosus: risks, mechanisms and therapeutic targets. Ann Rheumatic Dis. (2023) 82:999–1014. doi: 10.1136/ard-2022-223741 36792346

[71] Ambler WG Kaplan MJ . Vascular damage in systemic lupus erythematosus. Nat Rev Nephrol. (2024) 20:251–65. doi: 10.1038/s41581-023-00797-8 38172627 PMC11391830

[72] Brown GJ Cañete PF Wang H Medhavy A Bones J Roco JA . TLR7 gain-of-function genetic variation causes human lupus. Nature. (2022) 605:349–56. doi: 10.1038/s41586-022-04642-z 35477763 PMC9095492

[73] Gao L Slack M Barnas JL McDavid A Anolik J Looney RJ . Cell senescence in lupus. Curr Rheumatol Rep. (2019) 21:1. doi: 10.1007/s11926-019-0800-6 30637490 PMC6331499

[74] Lee YH Jung JH Seo YH Kim JH Choi SJ Ji JD . Association between shortened telomere length and systemic lupus erythematosus: a meta-analysis. Lupus. (2017) 26:282–8. doi: 10.1177/0961203316662721 27510600

[75] Pappa M Keramiotou K Sfikakis PP Tektonidou MG . Frailty is independently associated with subclinical cardiovascular disease in patients with systemic lupus erythematosus. Rmd Open. (2024) 10:e004527. doi: 10.1136/rmdopen-2024-004527 39313303 PMC11418478

[76] Wang X Diercks G Lambers WM Westra J Bootsma H Kroese FGM . Senescent progenitor cells in the skin of patients with cutaneous lupus erythematosus. J Invest Dermatol. (2022) 142:976–980.e2. doi: 10.1016/j.jid.2021.06.022 34273349

[77] Saito Y Yamamoto S Chikenji TS . Role of cellular senescence in inflammation and regeneration. Inflamm Regen. (2024) 44:28. doi: 10.1186/s41232-024-00342-5 38831382 PMC11145896

[78] Duan R Fu Q Sun Y Li Q . Epigenetic clock: a promising biomarker and practical tool in aging. Ageing Res Rev. (2022) 81:101743. doi: 10.1016/j.arr.2022.101743 36206857

[79] Mavrommatis C Belsky DW Ying K Moqri M Campbell A Richmond A . An unbiased comparison of 14 epigenetic clocks in relation to 174 incident disease outcomes. Nat Commun. (2025) 16:11164. doi: 10.1038/s41467-025-66106-y 41402269 PMC12708718

[80] Chervova O Panteleeva K Chernysheva E Widayati TA Florjanic Baronik Ž Hrbková N . Breaking new ground on human health and well-being with epigenetic clocks: a systematic review and meta-analysis of epigenetic age acceleration associations. Ageing Res Rev. (2024) 102:102552. doi: 10.1016/j.arr.2024.102552 39423872

[81] Gensous N Blanco P Lazaro E Mercié P Pellegrin I Richez C . Pilot study on accelerated aging in lupus using epigenetic biomarkers of age. Lupus. (2023) 32:129–35. doi: 10.1177/09612033221130976 36179673

[82] Haque S Rakieh C Marriage F Ho P Gorodkin R Teh LS . Brief report: shortened telomere length in patients with systemic lupus erythematosus. Arthritis And Rheumatism. (2013) 65:1319–23. doi: 10.1002/art.37895 23400670

[83] Honda M Mengesha E Albano S Nichols WS Wallace DJ Metzger A . Telomere shortening and decreased replicative potential, contrasted by continued proliferation of telomerase-positive CD8+CD28lo T cells in patients with systemic lupus erythematosus. Clin Immunol. (2001) 99:211–21. doi: 10.1006/clim.2001.5023 11318593

[84] Lai ZW Kelly R Winans T Marchena I Shadakshari A Yu J . Sirolimus in patients with clinically active systemic lupus erythematosus resistant to, or intolerant of, conventional medications: a single-arm, open-label, phase 1/2 trial. Lancet. (2018) 391:1186–91. doi: 10.1016/S0140-6736(18)30485-9 29551338 PMC5891154

[85] Yin Y Choi SC Xu Z Perry DJ Seay H Croker BP . Normalization of CD4^+^ T cell metabolism reverses lupus. Sci Transl Med. (2015) 7:2026–02-13. doi: 10.1126/scitranslmed.aaa0835 25673763 PMC5292723

[86] Manolakou T Nikolopoulos D Gkikas D Filia A Samiotaki M Stamatakis G . ATR-mediated DNA damage responses underlie aberrant B cell activity in systemic lupus erythematosus. Sci Adv. (2022) 8:eabo5840. doi: 10.1126/sciadv.abo5840 36306362 PMC9616496

[87] Zhu H Chen J Liu K Gao L Wu H Ma L . Human PBMC scRNA-seq–based aging clocks reveal ribosome to inflammation balance as a single-cell aging hallmark and super longevity. Sci Adv. (2023) 9:eabq7599. doi: 10.1126/sciadv.abq7599 37379396 PMC10306289

[88] Chung MKY Gong L Kwong DLW Lee VHF Lee AWM Guan XY . Functions of double-negative B cells in autoimmune diseases, infections, and cancers. EMBO Mol Med. (2023) 15:e17341. doi: 10.15252/emmm.202217341 37272217 PMC10493577

[89] Hu H Zhang G Chen T Liu Y Meng L Holmdahl R . Immunosenescence in autoimmune diseases. Autoimmun Rev. (2025) 24:103805. doi: 10.1016/j.autrev.2025.103805 40132774

[90] Furman D Campisi J Verdin E Carrera-Bastos P Targ S Franceschi C . Chronic inflammation in the etiology of disease across the life span. Nat Med. (2019) 25(12):1822–1832. doi: 10.1038/s41591-019-0675-0 31806905 PMC7147972

[91] Wu F Mu WC Markov NT Fuentealba M Halaweh H Senchyna F . Immunological biomarkers of aging. J Immunol. (2025) 214:889–902. doi: 10.1093/jimmun/vkae036 40443365 PMC12123219

[92] Higdon LE Gustafson CE Ji X Sahoo MK Pinsky BA Margulies KB . Association of premature immune aging and cytomegalovirus after solid organ transplant. Front Immunol. (2021) 12:661551. doi: 10.3389/fimmu.2021.661551 34122420 PMC8190404

[93] Eggenhuizen PJ Cheong RMY Lo C Chang J Ng BH Ting YT . Smith-specific regulatory T cells halt the progression of lupus nephritis. Nat Commun. (2024) 15:899. doi: 10.1038/s41467-024-45056-x 38321013 PMC10847119

[94] Han X Jiang Y Gu S Shen Y Ding H Chen S . IL-2 signalling sustains pathogenic age-associated B cell homeostasis in lupus. Ann Rheumatic Dis. (2026) 85:328–38. doi: 10.1016/j.ard.2025.08.012 40940283

[95] Qiu X Wen RF Wu F Mao J Azad T Wang Y . The role of double-negative B cells in the pathogenesis of systemic lupus erythematosus. Autoimmun Rev. (2025) 24:103821. doi: 10.1016/j.autrev.2025.103821 40274006

[96] Karimian K Groot A Huso V Kahidi R Tan KT Sholes S . Human telomere length is chromosome end– specific and conserved across individuals. Science. (2024) 384:533–9. doi: 10.1126/science.ado0431 38603523 PMC12425158

[97] Yu HJ Byun YH Park CK . Techniques for assessing telomere length: a methodological review. Comput Struct Biotechnol J. (2024) 23:1489–98. doi: 10.1016/j.csbj.2024.04.011 38633384 PMC11021795

[98] Teschendorff AE Horvath S . Epigenetic ageing clocks: statistical methods and emerging computational challenges. Nat Rev Genet. (2025) 26:350–68. doi: 10.1038/s41576-024-00807-w 39806006

[99] Fanouriakis A Kostopoulou M Andersen J Aringer M Arnaud L Bae SC . EULAR recommendations for the management of systemic lupus erythematosus: 2023 update. Ann Rheumatic Dis. (2024) 83:15–29. doi: 10.1136/ard-2023-224762 37827694

[100] Parodis I Lindblom J Toro-Domínguez D Beretta L Borghi MO Castillo J . Interferon and B-cell signatures inform precision medicine in lupus nephritis. Kidney Int Rep. (2024) 9:1817–35. doi: 10.1016/j.ekir.2024.03.014 38899167 PMC11184261

[101] Humrich JY Cacoub P Rosenzwajg M Pitoiset F Pham HP Guidoux J . Low-dose interleukin-2 therapy in active systemic lupus erythematosus (LUPIL-2): a multicentre, double-blind, randomised and placebo-controlled phase II trial. Ann Rheumatic Dis. (2022) 81:1685–94. doi: 10.1136/ard-2022-222501 35973803

[102] Wang W He S Zhang W Zhang H DeStefano VM Wada M . BCMA-CD19 compound CAR T cells for systemic lupus erythematosus: a phase 1 open-label clinical trial. Ann Rheumatic Dis. (2024) 83:1304–14. doi: 10.1136/ard-2024-225785 38777376

[103] Müller F Taubmann J Bucci L Wilhelm A Bergmann C Völkl S . CD19 CAR T-cell therapy in autoimmune disease — a case series with follow-up. N Engl J Med. (2024) 390:687–700. doi: 10.1056/NEJMoa2308917 38381673

[104] Farge D Biard L Weil B Girault V Lansiaux P Munia I . Allogeneic umbilical cord-derived mesenchymal stromal cells as treatment for systemic lupus erythematosus: a single-centre, open-label, doseescalation, phase 1 study. Lancet Rheumatol. (2025) 7:e261–73. doi: 10.1016/S2665-9913(24)00298-4 39706212

[105] Zhao C Chu Y Liang Z Zhang B Wang X Jing X . Low dose of IL-2 combined with rapamycin restores and maintains the long-term balance of Th17/treg cells in refractory SLE patients. BMC Immunol. (2019) 20:32. doi: 10.1186/s12865-019-0305-0 31484501 PMC6727508

[106] Alexander T Thiel A Rosen O Massenkeil G Sattler A Kohler S . Depletion of autoreactive immunologic memory followed by autologous hematopoietic stem cell transplantation in patients with refractory SLE induces long-term remission through de novo generation of a juvenile and tolerant immune system. Blood. (2009) 113(1):214–223. doi: 10.1182/blood-2008-07-168286 18824594

[107] Doglio M Alexander T Del Papa N Snowden JA Greco R . New insights in systemic lupus erythematosus: from regulatory T cells to CAR-T-cell strategies. J Allergy Clin Immunol. (2022) 150:1289–301. doi: 10.1016/j.jaci.2022.08.003 36137815

[108] Huang X Chen W Ren G Zhao L Guo J Gong D . Autologous hematopoietic stem cell transplantation for refractory lupus nephritis. Clin J Am Soc Nephrol. (2019) 14:719–27. doi: 10.2215/CJN.10570918 30979713 PMC6500938

[109] Ramsköld D Parodis I Lakshmikanth T Sippl N Khademi M Chen Y . B cell alterations during BAFF inhibition with belimumab in SLE. EBioMedicine. (2019) 40:517–27. doi: 10.1016/j.ebiom.2018.12.035 30593436 PMC6412067

[110] McCluskey D Shipa MRA Chowdhury K James JA Cooney LA Ehrenstein MR . IgA2+ B cells and IgA2 antidsDNA antibodies are selectively targeted by belimumab after rituximab therapy in systemic lupus erythematosus. Cell Rep Med. (2025) 6:102247. doi: 10.1016/j.xcrm.2025.102247 40706590 PMC12432362

[111] Müller F Hagen M Wirsching A Kharboutli S Aigner M Völkl S . CD19 CAR-T cells for treatment-refractory autoimmune diseases: the phase 1/2 CASTLE basket trial. Nat Med. (2026) 32:1142–51. doi: 10.1038/s41591-025-04185-6 41501497 PMC13004673

[112] Mackensen A Müller F Mougiakakos D Böltz S Wilhelm A Aigner M . Anti-CD19 CAR T cell therapy for refractory systemic lupus erythematosus. Nat Med. (2022) 28:2124–32. doi: 10.1038/s41591-022-02017-5 36109639

[113] Rao M Mikdashi J . A framework to overcome challenges in the management of infections in patients with systemic lupus erythematosus. Open Access Rheumatology: Res Rev. (2023) 15:125–37. doi: 10.2147/OARRR.S295036 37534019 PMC10391536

[114] Kubo S Nakayamada S Miyazaki Y Todoroki Y Ueno M Kusaka K . POS0192 lupus patients can be categorized into four groups based on immunophenotype, each exhibiting distinct relapse rates. Ann Rheum Dis. (2024) 83:403–404. doi: 10.1136/annrheumdis-2024-eular.2153

[115] He J Zhang R Shao M Zhao X Miao M Chen J . Efficacy and safety of low-dose IL-2 in the treatment of systemic lupus erythematosus: a randomised, double-blind, placebo-controlled trial. Ann Rheum Dis. (2020) 79(1):141–149. doi: 10.1136/annrheumdis-2019-215396 PMC6937406 31537547

[116] Pemberton SE Jackson SW . Lupus interference with B cell tolerance across the developmental continuum. Arthritis Rheumatol. (2023) 75:1503–5. doi: 10.1002/art.42485 36862382 PMC10474238

[117] Moysidou E Lioulios G Christodoulou M Xochelli A Stai S Iosifidou M . Increase in double negative B lymphocytes in patients with systemic lupus erythematosus in remission and their correlation with early differentiated T lymphocyte subpopulations. Curr Issues Mol Biol. (2023) 45:6667–81. doi: 10.3390/cimb45080421 37623240 PMC10453294

[118] Yu H Nagafuchi Y Fujio K . Clinical and immunological biomarkers for systemic lupus erythematosus. Biomolecules. (2021) 11:928. doi: 10.3390/biom11070928 34206696 PMC8301935

[119] Kaul A Gordon C Crow MK Touma Z Urowitz MB Ruiz-Irastorza G . Systemic lupus erythematosus. Nat Rev Dis Primers. (2016) 2:16039. doi: 10.1038/nrdp.2016.39 27306639

[120] Jenks SA Cashman KS Woodruff MC Lee FEH Sanz I . Extrafollicular responses in humans and SLE. Immunol Rev. (2019) 288:136–48. doi: 10.1111/imr.12741 30874345 PMC6422038

